# Use of a Computed Tomography Based Approach to Validate Noninvasive Devices to Measure Rotational Knee Laxity

**DOI:** 10.1155/2015/705201

**Published:** 2015-11-22

**Authors:** Simon Neumann, Stefan Maas, Danièle Waldmann, Pierre-Louis Ricci, Arno Zürbes, Pierre-Jean Arnoux, Frédéric Walter, Jens Kelm

**Affiliations:** ^1^Research Unit in Engineering Science, University of Luxembourg, 1359 Kirchberg, Luxembourg; ^2^Fachhochschule Bingen, 55411 Bingen am Rhein, Germany; ^3^Laboratoire de Biomécanique Appliquée, Université de la Méditerranée, 13916 Marseille, France; ^4^Clinique d'Eich, Centre Hospitalier de Luxembourg, 1460 Eich, Luxembourg; ^5^Chirurgisch-Orthopädisches Zentrum, Illingen, 66557 Saarland, Germany; ^6^Klinik für Orthopädie und Orthopädische Chirurgie, Universitätsklinikum des Saarlandes, 66424 Homburg/Saar, Germany

## Abstract

The purpose of this study is to validate a noninvasive rotational knee laxity measuring device called “Rotameter P2” with an approach based on Computed Tomography (CT). This CT-approach using X-rays is hence invasive and can be regarded as a precise reference method that may also be applied to similar devices. An error due to imperfect femur fixation was observed but can be neglected for small torques. The most significant estimation error is due to the unavoidable soft tissues rotation and hence flexibility in the measurement chain. The error increases with the applied torque. The assessment showed that the rotational knee angle measured with the Rotameter is still overestimated because of thigh and femur displacement, soft tissues deformation, and measurement artefacts adding up to a maximum of 285% error at +15 Nm for the Internal Rotation of female volunteers. This may be questioned if such noninvasive devices for measuring the Tibia-Femoral Rotation (TFR) can help diagnosing knee pathologies and investigate ligament reconstructive surgery.

## 1. Introduction

The knee is a voluminous and complex human joint [1, 2]. Located between the distal end of the femur and the proximal end of the tibia; it provides extension, flexion, and some rotations. It is the joint that bears during slow walking three times the body weight [3, 4]. A number of ligaments help controlling the movements and supply stability. The Anterior Cruciate Ligament (ACL) and the Posterior Cruciate Ligament (PCL), frequently torn in sporting activities, provide primarily sagittal plane stability but may also contribute to rotational stability.

Before treating these injuries by reconstructive surgery, a clinician carries out several manual tests to diagnose the degree of laxity. Those examinations such as the pivot shift or the dial test are performed by medical practitioners and depend therefore on their experience [5]. Several devices have been developed to quantify knee laxity and to make measurements more objective. But devices as the KT-1000 arthrometer [6] are limited to the anterior-posterior translation of the tibia, and studies call into question the intratester and intertester reliability of the device considered as a moderately reliable tool [7, 8]. Although reconstructive surgery permits sufficient repair of this ligament [9] in the sagittal plane, it remains limited in the restoration of rotational stability and no reliable easy-to-use device to measure knee rotation is available [10, 11].

**Figure 1:**
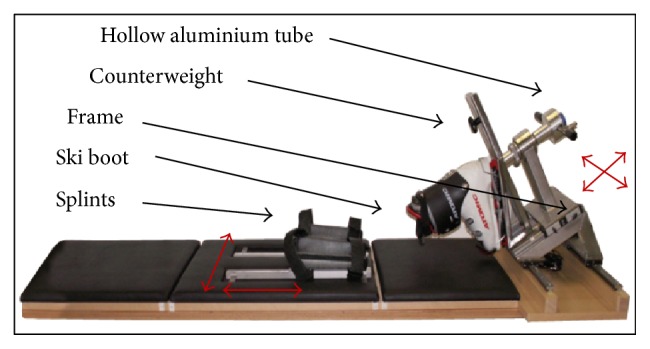
Rotameter P2 and its kinematics.

Such a device should measure noninvasively and independently of the user the Tibia-Femoral Rotation (TFR) in order to analyse rotational stability. It could allow extended trials with larger groups to investigate deeper the ligaments' role in rotational knee laxity. Moreover, it could aim to evaluate surgical reconstruction techniques as authors showed that tibia rotation is altered with ACL reconstruction after the rupture of a ligament [12, 13]. The purpose of this study is to evaluate and validate in vivo the second version of the Rotameter (P2) from a previous paper [14] where design, repeatability, and comparison with the literature data were presented. The CT-method as such is independent of this special noninvasive device and may be used to analyse similar measuring tools and allow comparison. Nevertheless, for a very short descriptive summary of this device, see Figure 1.

**Figure 2:**
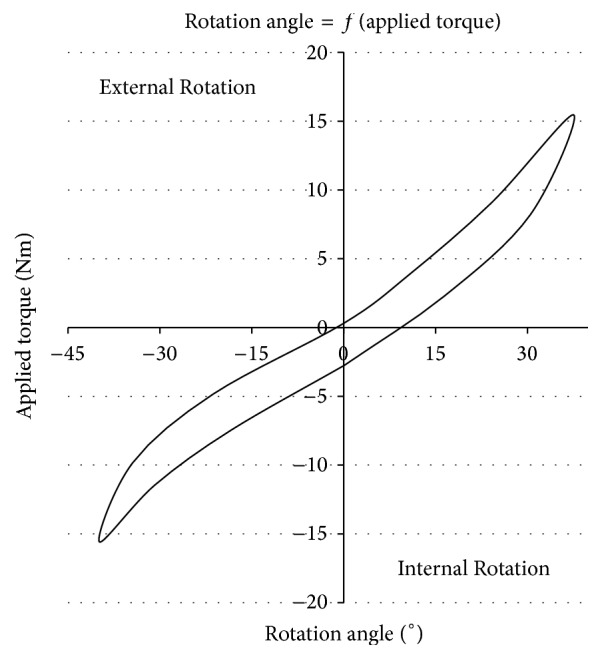
Characteristic curve of the patient's knee.

The subject puts on a ski boot with a snug fit and lies down in prone position with the knee flexed at 30°. The ski boot is attached to the frame with a normal ski-binding and a torque is manually applied with a handle. The torque is measured by strain gauges while the rotational angle is registered by an inclinometer. With these continuous measurements, a two-dimensional torque angle graph with hysteresis is plotted to illustrate the patient's rotational knee laxity versus the external loading (Figure 2).

For precision reasons, the frame is made of welded stainless steel with sufficient stiffness. The frame and the splints for fixing the thigh of the patient are simply adaptable to the individual's size and permit an easy but correct use. The error of repeatability was found to be inferior to 6° at ±15 Nm torque [14].

By comparison with a standard CT-method, the present paper aims at quantifying soft tissue movements and the total measuring error of P2. The use of X-rays appears to be a reliable method [15, 16] to measure the angulation between femur and tibia because bony structures can be clearly identified. It will therefore be considered as the standard measurement technique.

## 2. Methods

The initial Rotameter P2 [14] has been slightly modified to make it compatible with the whole body CT scanner (SOMATOM Emotion 6 from SIEMENS, Erlangen, Germany). First of all, its floor dimensions were reduced to avoid any contact with the scanner's ring. Then, a locking system was implemented to apply a defined torque, lock the handle, and thus keep the position while performing the CT.

Six healthy adult volunteers (three men and three women at the age of 25 ± 6, height of 174 ± 12 cm, and a weight of 75 ± 15 kg) with a normal body mass index and without any specific knee problems took part in these trials. Every subject underwent a Lachman and a pivot shift test by an experienced physician to exclude any kind of knee damage or impairment.

**Figure 3:**
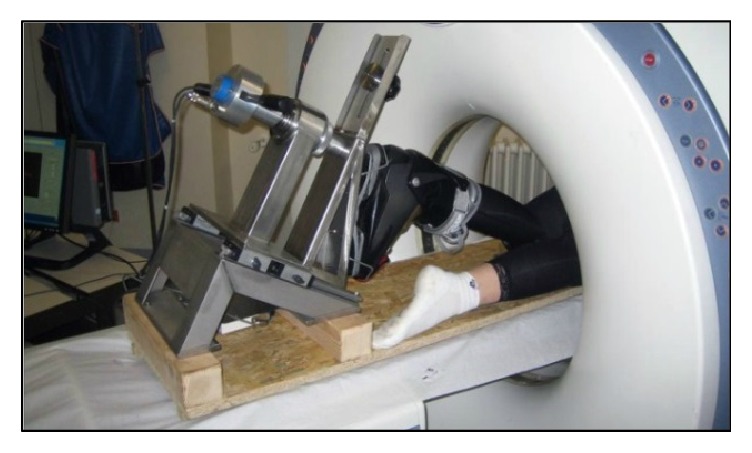
CT scanner with a patient attached to the adapted Rotameter P2.

To perform the trial as illustrated in Figure 3, the volunteer put on the best fitting ski boot and lay down in prone position. Then, his foot was fixed to the frame via the ski blade whereas the distal part of his thigh was attached to the assembly by means of adjustable splints and two Velcro belts.

In order to ensure the alignment of the lower limb (i.e., tibia and femur) and to relax muscles, two preliminary cycles with loading phases reaching ±15 Nm were done with imaging switched off. The full cycle was subdivided into four steps: (1) loading up to +15 Nm for the Internal Rotation, (2) returning to 0 Nm by unloading, (3) loading up to −15 Nm for the External Rotation, and (4) returning to 0 Nm by unloading again.

**Figure 4:**
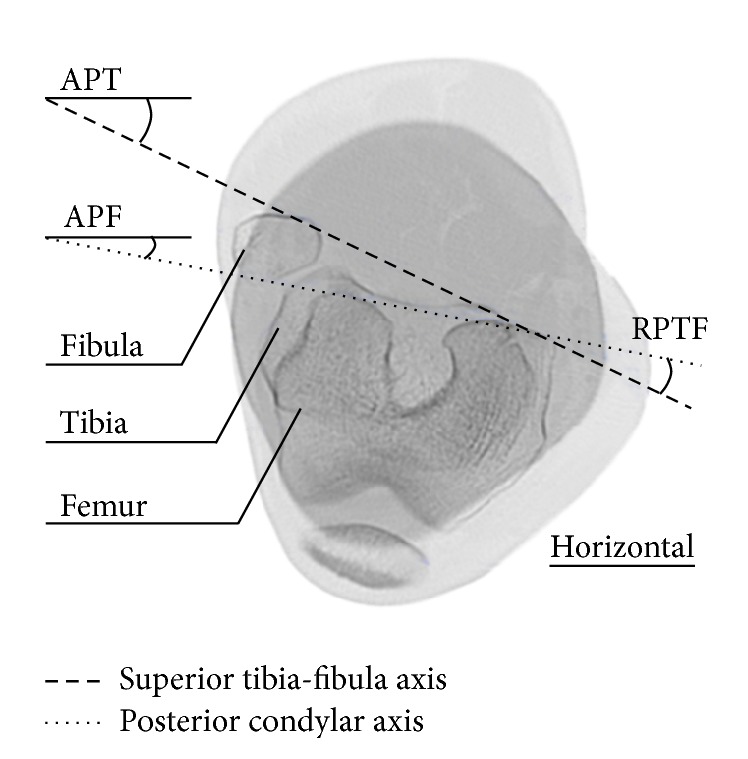
Tomographic axial slice of the lower limb.

The study was approved by the “Comité National d'Ethique et de Recherche” (CNER notice number 201201/03). But to limit the effective radiation dose as imposed by the Research board, only five pictures were taken per subject, although the patients underwent seven different values of applied torque (0 Nm, ±5 Nm, ±10 Nm, and ±15 Nm) which represented seven rotational angles of their lower limb. For the same reason, only 10 cm of the proximal tibia and 10 cm of the distal femur were considered, representing 20 cm of the joint. An example of a picture obtained at a certain torque value is given in Figure 4 with some abbreviations used for calculating different angles.

Several values defining the lower limb rotation were measured for every slice, and two axes were introduced: a dashed line named the “superior tibia-fibula axis” and a dotted one called the “posterior condylar axis” [17]. These lines, in conjunction with the horizontal, form the Absolute Position of the Tibia (APT) and the Absolute Position of the Femur (APF), both expressed in degrees (°). For a torque *i*, the difference between those two is the Relative Position of the Tibia with respect to the Femur (RPTF):

(1) $$\begin{array}{c} {\text{RPTF}}_{i}={\text{APT}}_{i}-{\text{APF}}_{i}. \end{array}$$

The Rotation of the Tibia relative to the Femur (RTF) is then calculated by comparing the actual configuration RPTF_*i*_ with the initial situation RPTF_0_:

(2) $$\begin{array}{c} {\text{RTF}}_{i}={\text{RPTF}}_{i}-{\text{RPTF}}_{0}. \end{array}$$

A summary of the measurements carried out on female volunteers (FVs) and male volunteers (MVs) with CT images is presented in Table 1 for the rest position and both the External Rotation (ER) and Internal Rotation (IR). Given that only five pictures were allowed by the Research board per subject, a repartition was chosen so that any torque value was measured four times, two with both genders of volunteers. The maximum torque was limited to ±15 Nm to protect the subjects from pain.

## 3. Results

The real TFR measured by CT images is hence

(3) $$\begin{array}{c} {\text{CT}}_{i}={\text{RTF}}_{i}. \end{array}$$

The TFR measured by CT images plus the rotation of the femur with respect to the support defines CTFD. The imperfect fixation of the femur caused its rotation and was called Femoral Deviation (FD):

(4) CTFDi=RTFi+FDiwith FDi=APFi−APF0.

The TFR measured by the Rotameter is simply denominated P2.  Table 2 gives the rotational angles (CT, CTFD, and P2) for the trials, as well as an average of the values of female subjects (AVG♀), male subjects (AVG♂), and all six volunteers (AVG).

**Figure 5:**
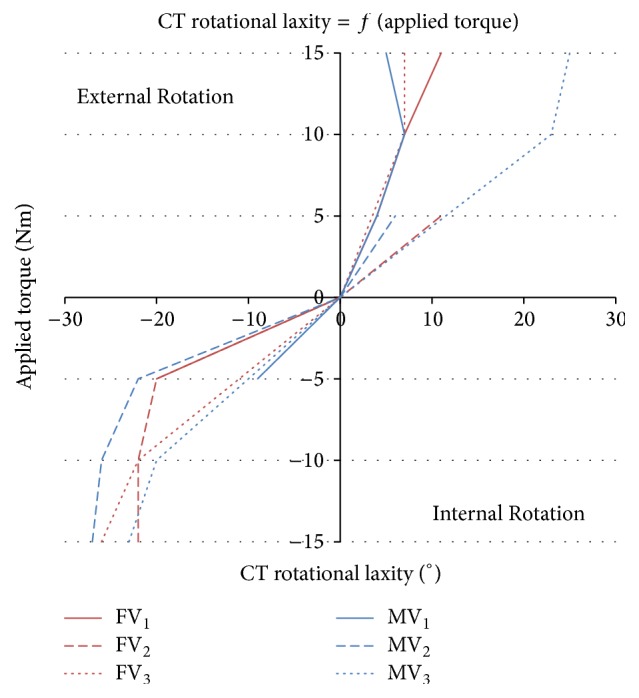
Torque angle graphs of individual subjects measured by CT.

Figure 5 represents graphically CT values of Table 2 according to the applied torque for each subject. ER amplitude is of the same order of magnitude for both genders and subjects. A similar evolution can be observed in IR, except for MV_3_ who had a sport training followed by stretching exercises prior to the clinical examination that might have altered the results.

**Figure 6:**
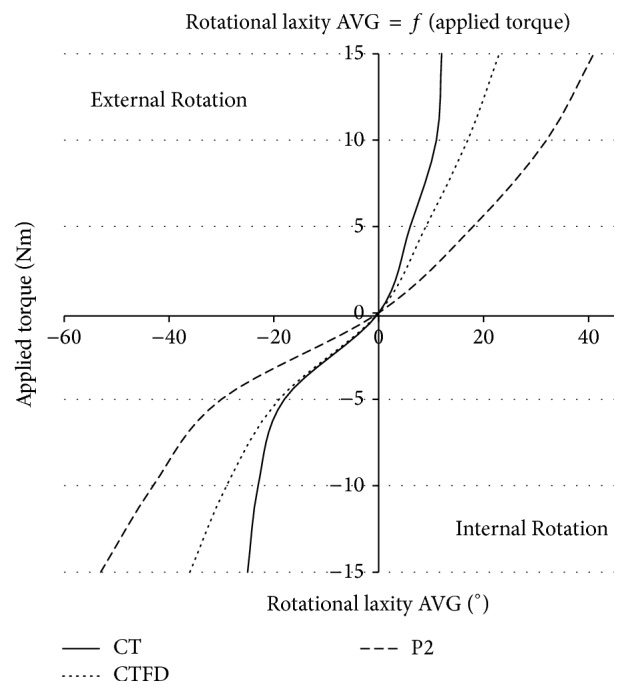
Torque angle graphs representing the average (AVG) of patients' rotational laxities measured by CT and P2.

Comparing the trials performed with P2 and the CT scanner reveals an overestimation of the rotational laxity when using the prototype. Additionally, the parallelism between CTFD and CT illustrates the presence of Femoral Deviation. Both divergences are increasing with the torque level.  Figure 6 is a graphical presentation of the average values (AVG) of Table 2 highlighting these observations.

The comparison of data between women and men (AVG♀ versus AVG♂) provided by available subjects reveals that there is no major difference between the real TFR represented by CT values and CTFD values in Table 2. But the values of P2 are considerably higher for female subjects than for male subjects, especially in case of ER.

To quantify the relative error for the TFR, Table 3 shows the relative deviation of femur, the Femoral Error (FE), and the Total Error (TE) of Rotameter P2 according to the following definitions:

(5) FE=CTFD−CTCT;TE=P2−CTCT.

In general, Femoral Error and Total Error are increasing with the torque. The IR error is approximately three times higher than the ER error for both genders. Additionally, errors in women are twice as high as errors in men. Apart from women having a Total Error reaching 285% in IR, the other values of Total Error are around 100%. Furthermore, it has to be considered that these errors were calculated with respect to the absolute TFR.

## 4. Discussion

The objective of the Rotameter was to measure effectively and objectively the rotational knee laxity in a noninvasive way. In a previous publication [14], the design process was deployed, the intertester and intratester reliability was shown, and the results were compared with the literature. In this study, an assessment was done by using CT scans to evaluate the measurement error of P2. Despite radiation exposure of the test persons and its complexity in operating, computerised navigation was chosen as a reference because of its precision in measuring rotational laxity [18, 19].

It was shown that the increasing inaccuracy of P2 was not only due to soft tissues, muscle activity, and measurement artefacts [20–24], but also due to the displacement of the thigh bone. Considering female and male volunteers with an applied torque of ±5 Nm, the Femoral Error reaches 24%, a relatively low value compared to others. This observation shows that a minor rotation of the femur is recorded with this low applied torque. In fact, the rotational deviation of the femur is negligible when compared with the rotational angulation of the knee. However, when considering ±10 Nm and ±15 Nm, the influence of the thigh bone on P2 is more evident as it reaches Femoral Errors of 46% and 73%, respectively. When increasing the torque from ±5 Nm to ±15 Nm, an even more important displacement of the thigh bone is observed. Although the volunteer's thigh is fastened more securely by the second prototype than by the previous version by means of adaptable stainless metal splints and Velcro belts, it is not possible to avoid completely the rotation of the thigh involving a maximum Femoral Error of 73% with the higher torque. In any of these cases, Femoral Error is higher for female than for male subjects. This observation calls into question the fixation of the thigh. Because of morphological differences between genders, upper legs of female and male volunteers are not held in place with the same effectiveness. Additional extra care should be given to find a way to secure it firmly in order to reduce this deviation as much as possible. Moreover, restricting the mobility of the hip joint should be considered by fixing the pelvis, because this could affect the results.

This divergence caused by bone motion modified the accuracy of the Rotameter. Even if some authors assumed a variance between skin and bone up to 13° [25] while measuring the rotation during a gait analysis, reducing undoubtedly the device's abilities, a lot of the actual inaccuracy is coming from other sources. With this purpose, Total Error includes any kind of artefact impairing P2 accuracy such as movement of soft tissues, muscle activity, or adjacent joint mobility. Unlike the limited Femoral Deviation at ±5 Nm, Total Error affects the results as soon as a torque is applied, from 122% for the lower torque to 189% for the maximum one. Although rotational laxities measured by CT are of the same magnitude for both genders, TFR values obtained with P2 are higher in female volunteers leading to a lower Total Error for males. This observation also expresses a morphological variation between genders that modifies P2 measurements. Even if rotational laxities are higher in ER, Femoral and Total Errors are almost twice as important in the case of IR. This statement could be contrary to the first assumption.

Considering the curves from Figure 6 with their respective data from Table 2, it is possible to monitor the real TFR included in P2 measurements and to compare the data with CT. As CT and P2 curves illustrate, the combination of all these error factors resulted in a measured knee rotation increasing exponentially with the torque. These differences between the rotations show that the values of ER are more accurate than that of IR, a phenomenon illustrating directly the observations made when considering the Total Error.

*Limitation and Advantages*. The values have to be handled with care due to the low number of volunteers allowed by the Research board. It is hence not possible to generalise the results to a large cohort of patients. Thanks to the promising results obtained previously, it would be useful to extend this study to a larger group of subjects to see if it is possible to observe the same facts and draw similar conclusions. At least, the repeatability could be easily checked by carrying out an extended clinical study which is currently ongoing.

This prototype P2 was designed to minimise measurement and soft tissue errors. Therefore, attention was drawn on the boot's stiffness to limit ankle rotation and avoid foot deviation when applying the torque. Careful attention has also been taken to ensure a proper fixation of the femur by the use of two cladded cone-shaped half pipes mounted on adjustable splints for adjustments. Additionally, patient's comfort in a laying position should be further investigated to ensure maximum muscle relaxation. As a consequence, rotation resistant properties could be prevented, thus ensuring an adequate rotational knee laxity.

Errors may also occur by using the CT scan when drawing the axes on the pictures. First, because of a possible relative motion between proximal parts of the tibia and the fibula, errors resulting from this mobility are however considered negligible if compared to other ones thanks to rigid boots restricting the motion of the ankle. Secondly, defining the bones' boundaries is a task to be treated with care. Hence, the measurements were realised by an experienced radiologist within an accuracy of one degree, which is a negligibly small error compared to the estimations coming from femoral displacement or Total Error of P2. As all measurements were double checked, it was concluded that CT technique reflects the actual TFR of the patient with high precision. And even if this approach exposes subjects to small radiation, it was approved by the “Comité National d'Ethique et de Recherche” (CNER notice number 201201/03) because of its efficiency and its restricted number of subjects. Apart from small modifications on P2, the CT-approach developed in this study is easy to set up and can be used by other authors to evaluate the precision of their devices. It would allow the comparison between invasive [20, 21, 23] and noninvasive [20, 24] prototypes and disclose possibilities for further improvements.

## 5. Conclusion

The Rotameter was coupled to a gold standard CT scanner to measure precisely the TFR and to assess the precision of the developed device. On average, the P2 measuring error is approximately 150% higher than the real TFR, which is a quite important error. It was observed that the displacement of the thigh bone (1) is quite small for low torques. Although this error increases with the load, its value is less important than the approximation made by tissues, muscles' activity, and nearby joints' mobility (2) which start acting as soon as an external force is applied to the lower limb. The better correlation of ER compared to IR should be treated with care due to the restricted number of volunteers (*n* = 6). Further studies are necessary to develop this user friendly and noninvasive device in order to get closer to the actual TFR value. Additional research is necessary to reflect with accurateness patients' rotational knee laxity.

Some further constructive improvements on prototype P2 are still feasible and come in sight. An easy-to-use measurement device for TFR would be valuable for a lot of clinical studies if the qualitative information is really reliable though not absolutely correct.

## Figures and Tables

**Table 1:** Data achieved by CT scanner on female volunteers (FVs) and male volunteers (MVs).

Torque (Nm)	Rest	External *R* (°)			Internal *R* (°)		
	0	−5	−10	−15	+5	+10	+15
FV_1_							
APF	2	3			7	7	15
APT	−7	−26			2	5	17
RPTF	−9	−29			−5	−2	2
FV_2_							
APF	−7	−11	−17	−23	−8		
APT	−18	−42	−50	−56	−8		
RPTF	−11	−31	−33	−33	0		
FV_3_							
APF	−3		−7	−12		5	9
APT	−7		−33	−42		8	12
RPTF	−4		−26	−30		3	3
MV_1_							
APF	−7	−7			−5	−3	1.5
APT	−16	−25			−10	−5	−1.5
RPTF	−9	−18			−5	−2	−3
MV_2_							
APF	−1	−2	−6	−11	3		
APT	−9	−32	−40	−46	1		
RPTF	−8	−30	−34	−35	−2		
MV_3_							
APF	−9		−15	−21		−2	−1
APT	−25		−51	−60		5	8
RPTF	−16		−36	−39		7	9

APF: Absolute Position of the Femur; APT: Absolute Position of the Tibia; RPTF: Relative Position of the Tibia with respect to the Femur.

**Table 2:** Angular rotation achieved by CT and P2 on female volunteers (FVs) and male volunteers (MVs).

Torque (Nm)	Rest	External *R* (°)			Internal *R* (°)		
	0	−5	−10	−15	+5	+10	+15
FV_1_							
CT	0	−20			4	7	11
CTFD	0	−19			9	12	24
P2	0	−36			22	33	43
FV_2_							
CT	0	−20	−22	−22	11		
CTFD	0	−24	−32	−38	10		
P2	0	−41	−50	−60	20		
FV_3_							
CT	0		−22	−26		7	7
CTFD	0		−26	−35		15	19
P2	0		−47	−61		28	36
AVG♀							
CT	**0**	**−20**	**−22**	**−24**	**8**	**7**	**9**
CTFD	**0**	**−22**	**−29**	**−37**	**10**	**14**	**22**
P2	**0**	**−39**	**−49**	**−61**	**21**	**31**	**40**
MV_1_							
CT	0	−9			4	7	5
CTFD	0	−9			6	11	14
P2	0	−15			11	24	32
MV_2_							
CT	0	−22	−26	−27	6		
CTFD	0	−23	−31	−37	10		
P2	0	−27	−38	−45	17		
MV_3_							
CT	0		−20	−23		23	25
CTFD	0		−26	−35		30	33
P2	0		−36	−45		43	53
AVG♂							
CT	**0**	**−16**	**−23**	**−25**	**5**	**15**	**15**
CTFD	**0**	**−16**	**−29**	**−36**	**8**	**21**	**24**
P2	**0**	**−21**	**−37**	**−45**	**14**	**34**	**43**

AVG							
CT	**0**	**−18**	**−23**	**−25**	**6**	**11**	**12**
CTFD	**0**	**−19**	**−29**	**−36**	**9**	**17**	**23**
P2	**0**	**−30**	**−43**	**−53**	**18**	**32**	**41**

Tibia-Femoral Rotation (TFR) measured by using the following.

(i) CT: Computed Tomography.

(ii) CTFD: Computed Tomography including Femoral Deviation.

(iii) P2: Rotameter P2.

**Table 3:** Femoral Error and Total Error of P2 in percentage.

Torque (Nm)	Femoral Error (%)				Total Error (%)			
	5	10	15	AVG	5	10	15	AVG
ER								
FVs	8	32	52	**30**	93	120	152	**122**
MVs	3	24	44	**24**	35	61	80	**59**
IR								
FVs	27	93	139	**86**	180	336	339	**285**
MVs	60	37	57	**51**	180	123	183	**162**
AVG	**24**	**46**	**73**		**122**	**160**	**189**	

ER: External Rotation; IR: Internal Rotation.
